# Supplementary material for: Real-World Evidence on the Clinical Characteristics and Management of Patients with Chronic Lymphocytic Leukemia in Spain Using Natural Language Processing: The SRealCLL Study
Source: Cancers (Basel). 2023 Aug 10;15(16):4047. doi: 10.3390/cancers15164047 (PMC10452602; doi:10.3390/cancers15164047)
Supplement: Supplementary file 1 [file cancers-15-04047-s001.zip › cancers-2493619-supplementary.pdf]

Supplementary Materials

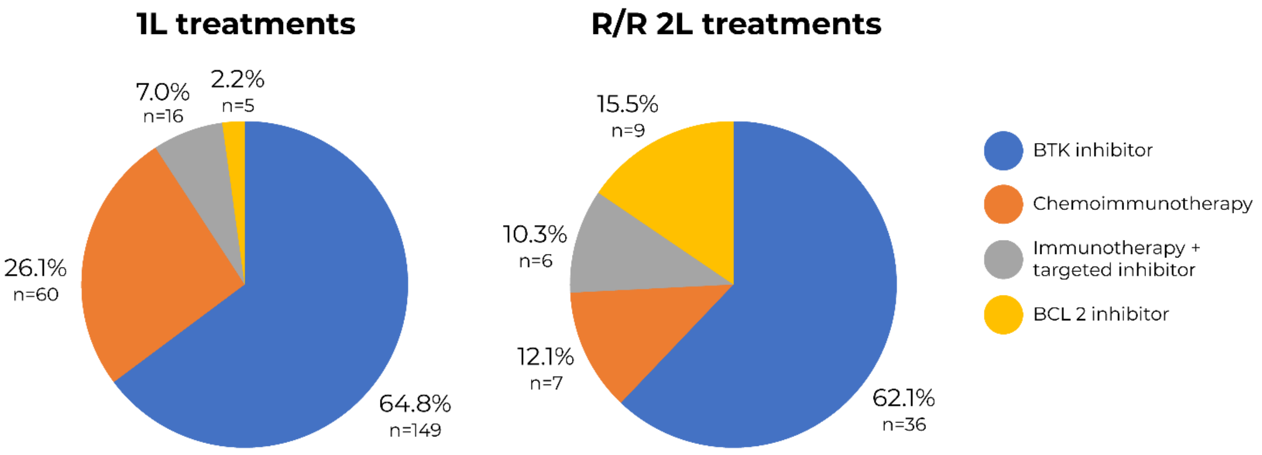

Supplementary Figure S1. Antineoplastic treatments for chronic lymphocytic leukemia grouped by mechanism of action.

Percentage (n) of patients from the 1L (left) or R/R 2L (right) treatment groups according to their mechanism of action. Treatments were detected from index date to the last follow up. The individual treatments in each group are as follows. *BTK inhibitor*: ibrutinib; *Chemoimmunotherapy:* bendamustine + rituximab, chlorambucil + rituximab, fludarabine + cyclophosphamide + rituximab, obinutuzumab + chlorambucil; *Immunotherapy + targeted inhibitor:* venetoclax + rituximab, ibrutinib + obinutuzumab, idelalisib + rituximab; BCL2 inhibitor: venetoclax.

1L: first-line; 2L: second-line; R/R: relapse/refractory.

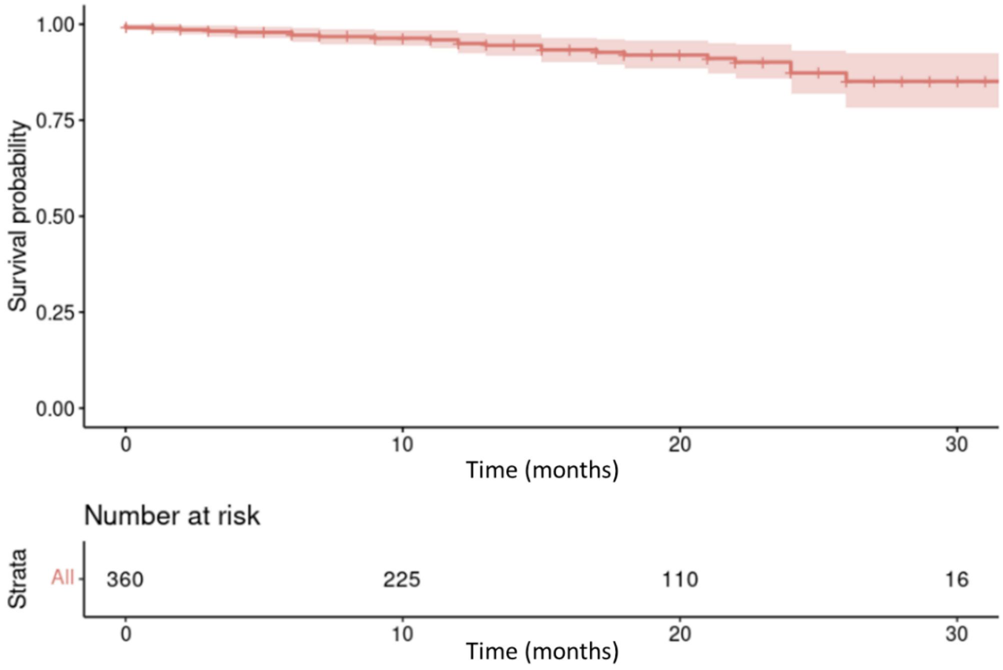

Supplementary Figure S2. Kaplan-Meier overall survival curve of all CLL patients diagnosed during the study period.

Supplementary Table S1. Evaluation metrics of *EHRead®* performance identifying key variables.

	Recall	Precision	F1-score
Chronic lymphocytic leukemia	0.987603	0.956	0.971545
Idelalisib	0.916667	0.982143	0.948276
Ibrutinib	0.904762	0.853933	0.878613
Obinutuzumab †	0.736842	0.293194	0.419476

† Reading of the term "obinutuzumab" was suboptimal, since the acronym "O" was included as a synonym of this variable, leading to many false positives during *EHRead®* detection.

Supplementary Table S2. Treatment switch matrix describing the number of patients switching from a specific first-line treatment (rows) to another second-line treatment (column).

1L treatment	2L treatment								
	BR	*RCI*	*FCR*	*OCI*	*IBR*	*VEN*	*VENR*	*IBRO*	*IDER*
BR	0	0	0	0	4	0	0	0	0
RCI	0	0	0	1	1	0	0	0	0
FCR	0	0	0	0	1	0	0	0	0
OCI	0	1	0	0	1	0	0	0	0
IBR	1	0	1	2	0	4	1	0	1
VEN	0	0	0	0	1	0	0	0	0
VENR	0	0	0	0	0	0	0	0	0
IBRO	0	0	0	0	0	0	0	0	0
IDER	0	0	0	0	3	1	0	0	0

Only treatment switches that have occurred during the study period are reflected in this matrix.
1L: first-line; 2L: second-line; BR: bendamustine+rituximab; FCR: fludarabine+cyclophosphamide+rituximab; IBR: ibrutinib; IBRO: ibrutinib+obinutuzumab; IDER: idelalisib+rituximab; OCI: obinutuzumab+chlorambucil; RCI: rituximab+chlorambucil; VEN: venetoclax; VENR: venetoclax+rituximab.
